# Beyond Sinonasal Undifferentiated Carcinoma: Molecular Reclassification of a SMARCB1-Deficient Sinonasal Carcinoma With Pulmonary Metastasis

**DOI:** 10.7759/cureus.117783

**Published:** 2026-10-11

**Authors:** Jean C Lafontaine-Álvarez, David E Barreto, Ricardo Fernandez-Gonzalez, Mauricio Caceres-Chacon, Maria S Correa Rivas

**Affiliations:** 1 Pathology and Laboratory Medicine, University of Puerto Rico School of Medicine, Medical Sciences Campus, San Juan, USA; 2 Pulmonary and Critical Care Medicine, San Juan City Hospital, San Juan, USA; 3 Diagnostic Radiology, University of Puerto Rico School of Medicine, Medical Sciences Campus, San Juan, USA

**Keywords:** immunohistochemistry, ini1 loss, metastatic sinonasal carcinoma, molecular profiling, molecular reclassification, pulmonary metastasis, sinonasal undifferentiated carcinoma, smarcb1-deficient sinonasal carcinoma, smarcb1 deletion, swi/snf complex

## Abstract

A 56-year-old man presented with a nine-month history of progressive left-sided nasal obstruction, epistaxis, facial and palatal pain, and proptosis. Imaging demonstrated a large destructive left sinonasal mass extending into the left maxillary sinus and orbit. An endoscopic biopsy revealed a poorly differentiated epithelial malignancy, initially classified as a sinonasal undifferentiated carcinoma (SNUC). Immunohistochemistry demonstrated strong cytokeratin expression and complete loss of integrase interactor 1 (INI1) nuclear expression in tumor cells, with retained staining in non-neoplastic elements.

The patient underwent a left subtotal maxillectomy with hard palate resection. The tumor measured at least 8 cm, involved the pterygoid muscle, and extended to the medial and superior resection margins. Following surgery, he received platinum-based chemotherapy and radiotherapy. Approximately two years later, he presented with progressive exertional dyspnea. Imaging revealed a large left pleural effusion and bilateral soft-tissue-attenuation pulmonary nodules. Pleural fluid evaluation was nondiagnostic for malignancy because of limited cellularity; however, a computed tomography-guided core biopsy of a right pulmonary nodule revealed metastatic carcinoma morphologically concordant with the sinonasal primary, with strong cytokeratin expression and persistent loss of INI1.

The patient subsequently received six cycles of pembrolizumab combined with cyclophosphamide, doxorubicin, and vincristine. Comprehensive molecular profiling of archived primary tumor tissue later identified a pathogenic SMARCB1 copy-number deletion, establishing the diagnosis of SMARCB1-deficient sinonasal carcinoma. At the last documented follow-up, fluorodeoxyglucose (FDG) positron emission tomography/computed tomography (PET/CT) demonstrated persistent metabolically active thoracic metastatic disease without interval progression or new sites of disease. This case illustrates how integration of morphology, immunohistochemistry, metastatic-site comparison, and molecular testing can refine the classification of a poorly differentiated sinonasal carcinoma initially diagnosed as SNUC.

## Introduction

High-grade sinonasal carcinomas comprise a heterogeneous group of malignant epithelial neoplasms with overlapping morphologic and immunophenotypic features. Historically, tumors lacking definitive squamous, glandular, or neuroendocrine differentiation were often classified as sinonasal undifferentiated carcinoma (SNUC), a diagnosis of exclusion. Advances in immunohistochemistry and molecular profiling have distinguished several biologically discrete entities or molecular subsets within this group, including nuclear protein in testis (NUT) carcinoma, isocitrate dehydrogenase 2 (IDH2)-mutant SNUC, and switch/sucrose nonfermentable (SWI/SNF) complex-deficient sinonasal carcinoma [1,2]. The latter category encompasses tumors characterized by deficiency of either SMARCB1 or SMARCA4 [2].

SMARCB1-deficient sinonasal carcinoma, a molecularly defined subtype of SWI/SNF complex-deficient sinonasal carcinoma, was first characterized as a distinct entity in 2014 [3,4]. It is characterized by complete loss of nuclear integrase interactor 1 (INI1) expression, the protein encoded by the tumor-suppressor gene SMARCB1, a core component of the SWI/SNF chromatin-remodeling complex. Histologically, these tumors frequently demonstrate basaloid or rhabdoid cytomorphology arranged in nests, sheets, or cords, often without specific squamous or glandular differentiation. Their morphologic heterogeneity and variable immunophenotype may lead to classification as SNUC or other poorly differentiated sinonasal malignancies, particularly with limited biopsy material [3-5]. Recognition of INI1 loss is therefore an important diagnostic clue that should prompt molecular correlation when available.

We present a SMARCB1-deficient sinonasal carcinoma initially classified as SNUC in a 56-year-old man who subsequently developed pulmonary metastatic disease. Complete loss of INI1 expression was present in the initial biopsy, resection specimen, and pulmonary metastasis, while comprehensive molecular profiling of the primary tumor identified a pathogenic SMARCB1 copy-number deletion. Although this reclassification did not alter the treatment already administered in this case, accurate identification has diagnostic and prognostic significance and may inform consideration of molecularly guided therapeutic strategies or clinical trials [6]. This case illustrates the value of integrating morphology, immunohistochemistry, comparison of primary and metastatic specimens, and molecular profiling to refine the classification and clinical assessment of poorly differentiated sinonasal carcinomas.

## Case presentation

A 56-year-old man with a medical history of essential hypertension treated with oral antihypertensives was evaluated for a nine-month history of progressive left-sided nasal obstruction, recurrent epistaxis, facial and palatal pain with paresthesia, headaches, rhinorrhea, and left-sided epiphora. Physical examination demonstrated mild left proptosis with preserved extraocular movements, bulging of the left hard palate, and a left nasal cavity mass draining serosanguineous fluid. No cervical lymphadenopathy was identified. Computed tomography (CT) demonstrated a large, locally invasive mass centered in the left nasal cavity, with significant osseous destruction and extension into the extraconal left orbit, maxillary sinus, ethmoidal air cells, and sphenoid sinus (Figure 1).

**Figure 1:**
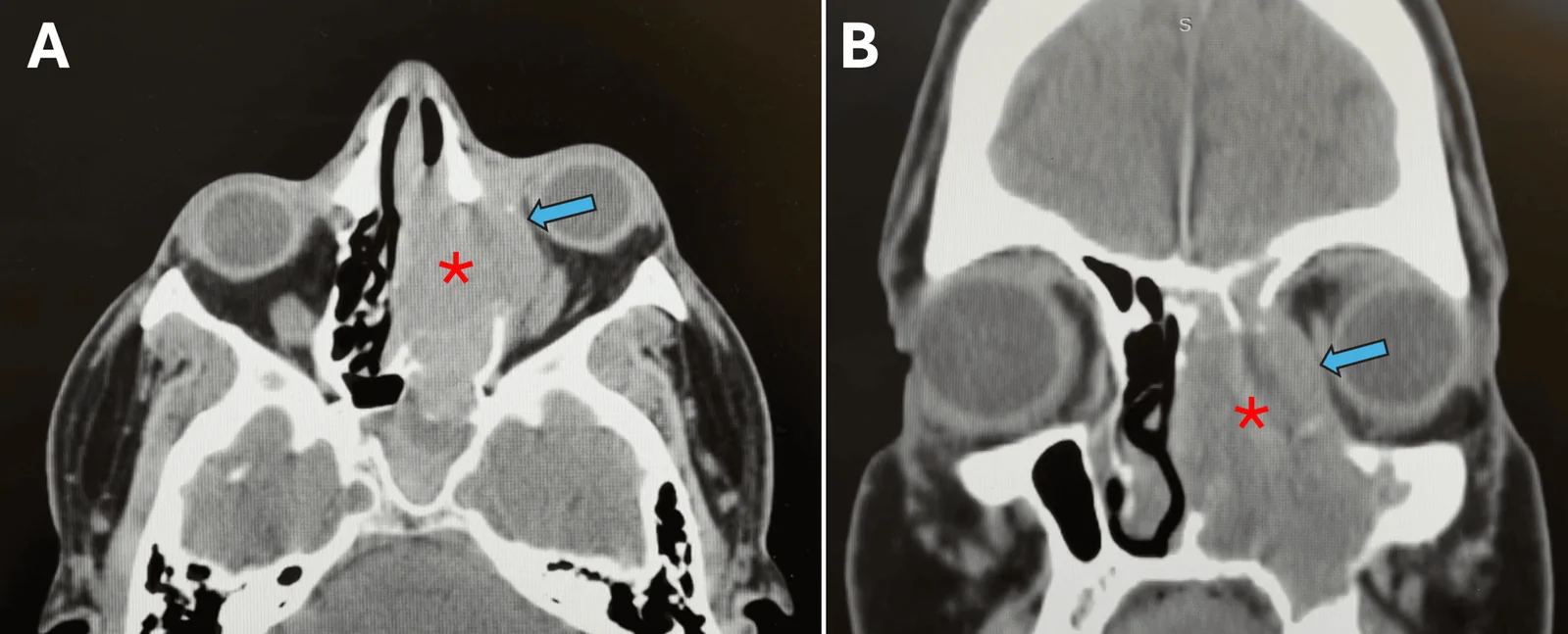
Contrast-enhanced computed tomography of the paranasal sinuses. Axial (A) and coronal (B) images demonstrate a large, locally destructive left sinonasal mass (red asterisk) centered in the nasal cavity, with destructive extension into the adjacent ipsilateral paranasal sinuses and left extraconal orbit (solid blue arrow).

Endoscopic examination revealed a highly vascular, rubbery left nasal cavity mass with focal cavitary areas. Multiple biopsies were obtained. Intraoperative consultation confirmed malignancy but deferred definitive classification pending immunohistochemical evaluation. Histologic examination demonstrated a poorly differentiated epithelial malignancy composed predominantly of basaloid cells arranged in solid sheets, with associated necrosis and stromal reaction (Figure 2).

**Figure 2:**
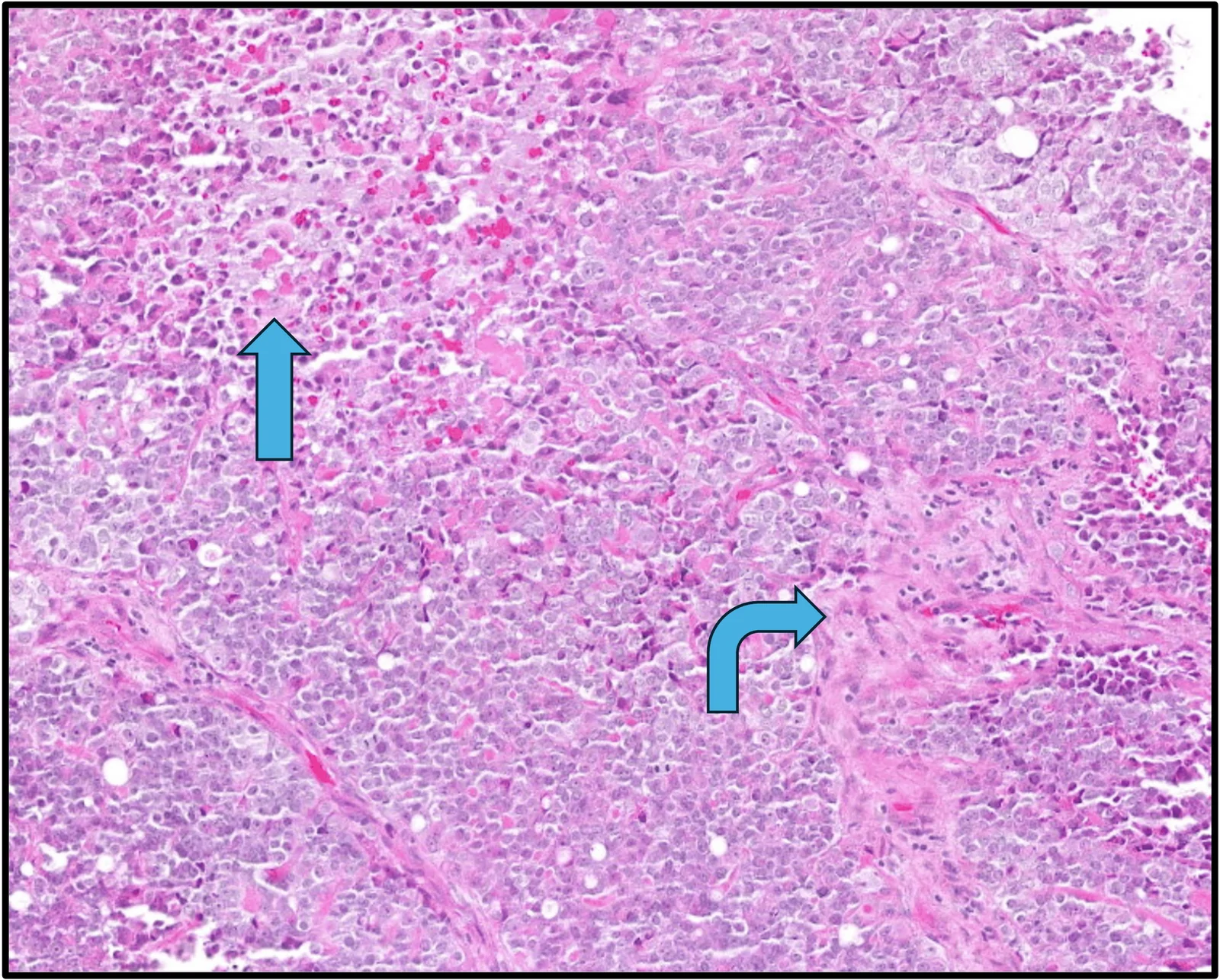
Histopathologic features of the primary sinonasal tumor. Hematoxylin and eosin staining demonstrates a poorly differentiated carcinoma arranged in solid nests and sheets, with associated tumor necrosis (straight blue arrow) and fibrous stromal septa (curved blue arrow) (20×).

The tumor showed strong, diffuse expression of cytokeratin 8/18, patchy pancytokeratin and cytokeratin 7 expression, strong, diffuse p16 staining, and focal expression of neuron-specific enolase. It was negative for cluster of differentiation (CD)45, CD99, chromogranin, synaptophysin, S100, cytokeratin 5/6, p40, p63, cellular-KIT (c-KIT), and Epstein-Barr virus-encoded RNA. INI1 immunohistochemistry demonstrated complete loss of nuclear expression in tumor cells, with retained staining in non-neoplastic stromal elements serving as an internal positive control (Figure 3).

**Figure 3:**
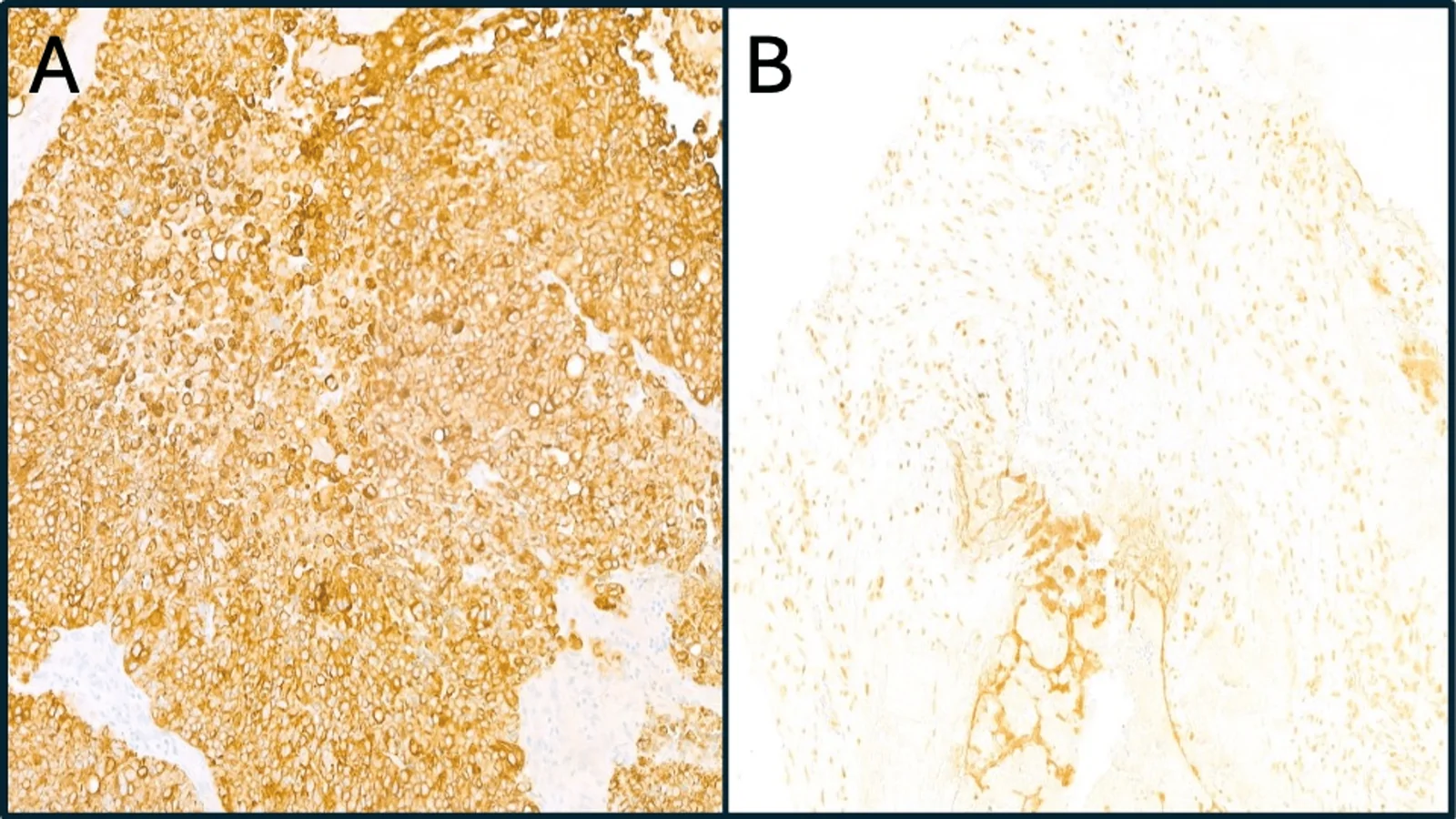
Immunohistochemical findings of the primary sinonasal tumor. (A) The tumor cells demonstrate strong, patchy pancytokeratin expression, confirming epithelial differentiation (20×). (B) INI1 immunohistochemistry shows complete loss of nuclear expression in the tumor cells, with retained nuclear staining in non-neoplastic stromal elements serving as an internal positive control (10×). INI1: integrase interactor 1.

Based on the morphologic and immunophenotypic findings available at the time, the lesion was initially classified as SNUC.

Approximately seven weeks after the biopsy, the patient underwent a left lateral rhinotomy with subtotal maxillectomy, hard palate resection, extranasal ethmoidectomy, sphenoidotomy, and resection of the tumor and pterygoid plates. The fragmented tumor measured at least 8 cm and extensively involved the left maxillary sinus. Histologic examination demonstrated an extensively invasive, poorly differentiated carcinoma with solid growth, geographic necrosis, and fibrotic stroma (Figure 4).

**Figure 4:**
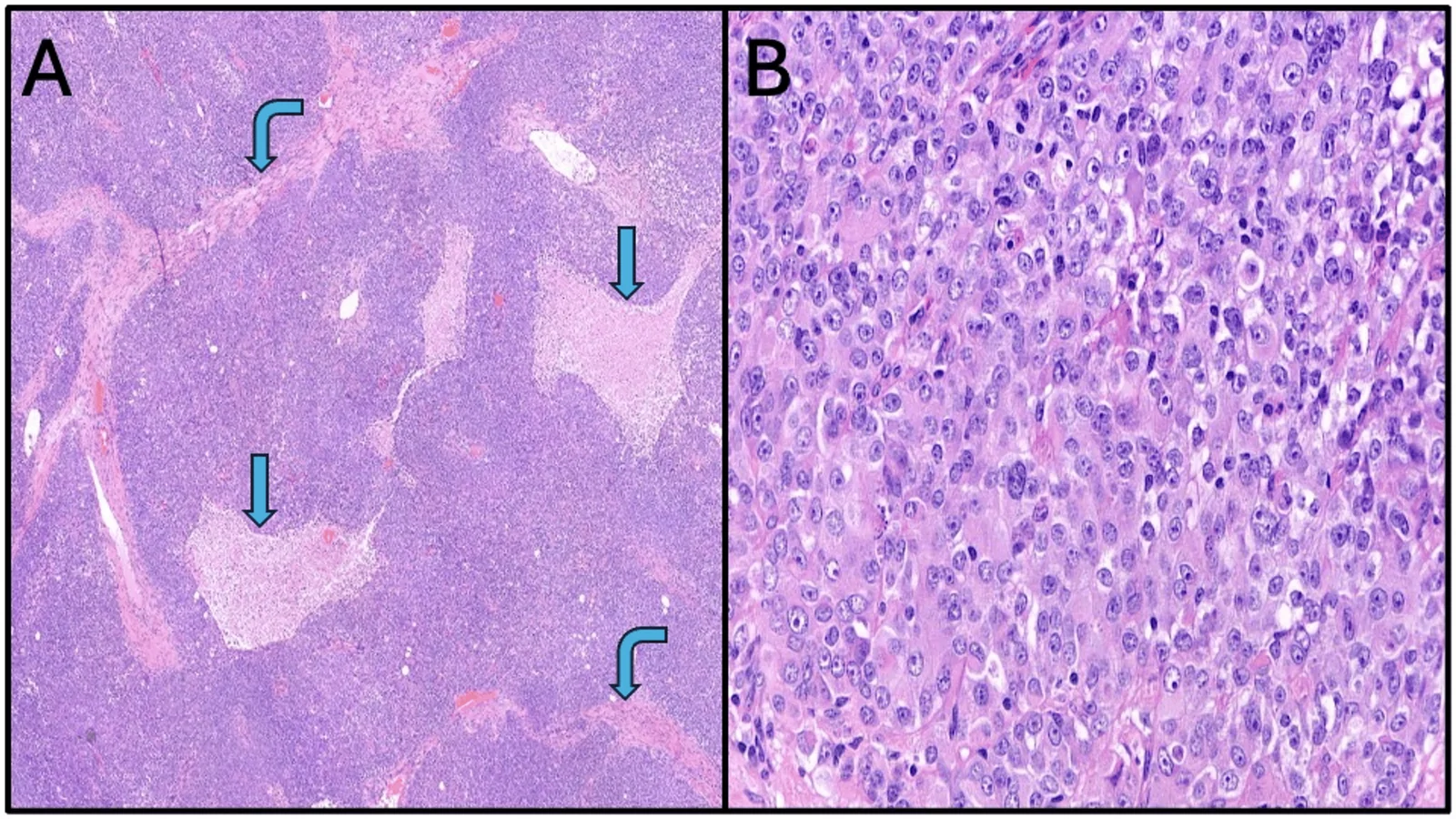
Histopathologic features of the primary tumor resection. (A) Low-power hematoxylin and eosin view showing carcinoma arranged in solid nests and sheets with confluent geographic necrosis (straight blue arrows) and intervening stromal fibrosis (curved blue arrows). (B) High-power hematoxylin and eosin view showing poorly differentiated epithelioid tumor cells with moderate eosinophilic cytoplasm, marked variation in nuclear size, irregular nuclear contours, vesicular chromatin, and conspicuous nucleoli. Occasional cells exhibit eccentrically located nuclei and more abundant eosinophilic cytoplasm, imparting focal rhabdoid features (40x).

Tumor involvement of the left pterygoid muscle was identified. The medial and superior resection margins were involved by carcinoma, while the separately submitted oral mucosa was negative for malignancy. Neither lymphovascular nor perineural invasion was identified, and no regional lymph nodes were submitted. Based on the nasal cavity and ethmoid sinus staging scheme used in the original pathology report, the tumor was assigned a pathologic category of pT3; the pathologic nodal category could not be assigned because no regional lymph nodes were submitted. INI1 immunohistochemistry again demonstrated complete loss of nuclear expression in the neoplastic cells, with retained staining in non-neoplastic elements serving as internal positive controls (Figure 5).

**Figure 5:**
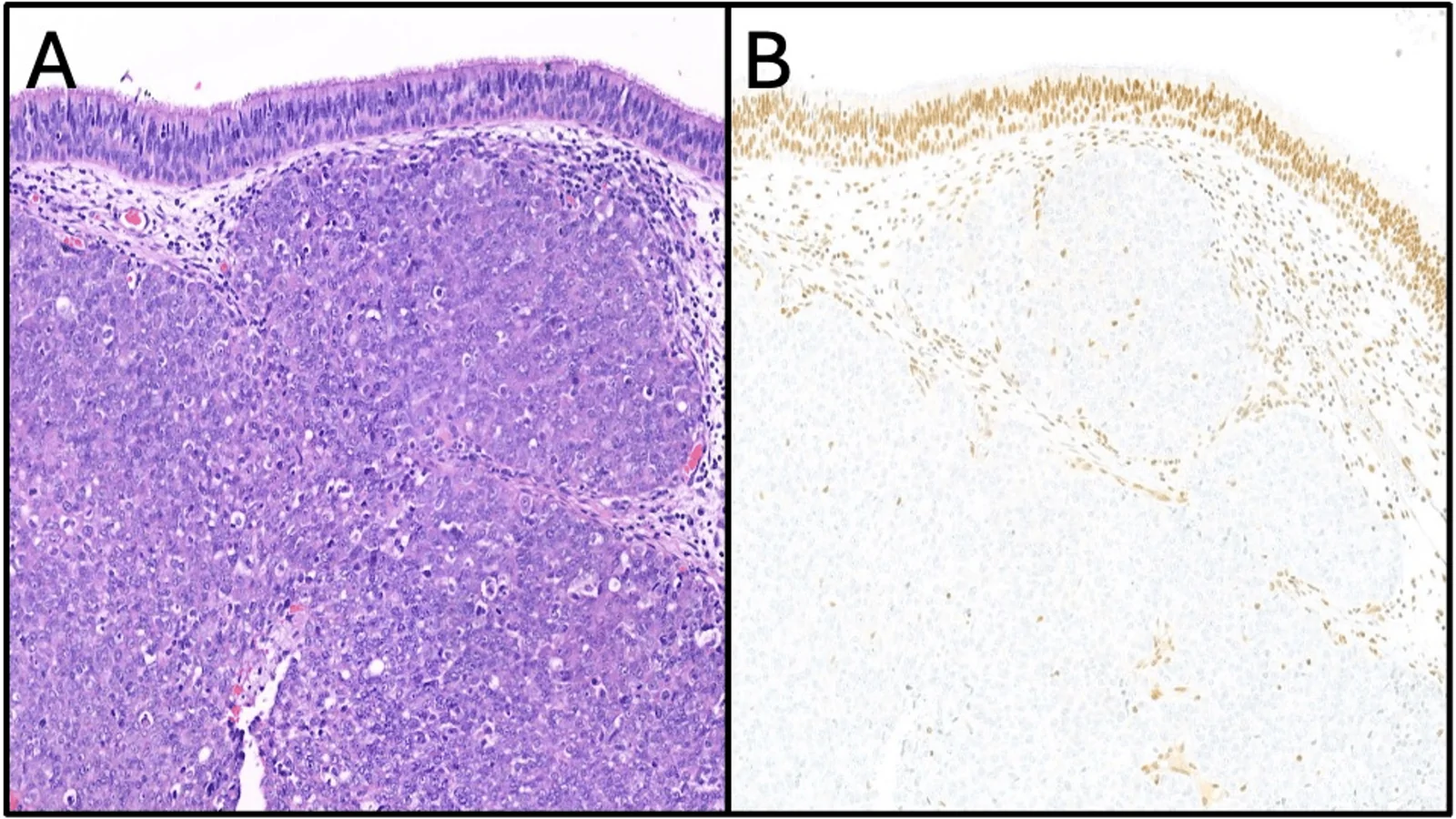
Loss of INI1 expression in the primary tumor resection. (A) The resection specimen demonstrates a poorly differentiated carcinoma arranged in solid subepithelial nests beneath intact respiratory mucosa (hematoxylin and eosin, 20×). (B) Corresponding INI1 immunohistochemistry shows complete loss of nuclear expression in the tumor cells, with retained nuclear staining in the overlying respiratory epithelium and stromal elements serving as internal positive controls (20×). INI1: integrase interactor 1.

Following surgery, the patient received adjuvant radiotherapy consisting of 40 fractions to a total dose of 7,200 cGy, along with nine cycles of platinum-based chemotherapy, comprising three cycles of cisplatin followed by six cycles of carboplatin. Treatment was completed approximately four months after surgery.

Approximately two years after the original diagnosis, the patient presented to the emergency department with progressive exertional dyspnea of two weeks’ duration. Chest radiography demonstrated a left pleural effusion, and subsequent CT confirmed a large left pleural effusion with bilateral pulmonary nodules, more prominent in the right lung (Figure 6A). Thoracentesis yielded an exudative effusion. Cytologic evaluation showed only rare atypical epithelial cells and was nondiagnostic for malignancy because of limited cellularity. A CT-guided core biopsy of a right pulmonary nodule was subsequently performed, with two tissue cores obtained from the targeted lesion (Figure 6B).

**Figure 6:**
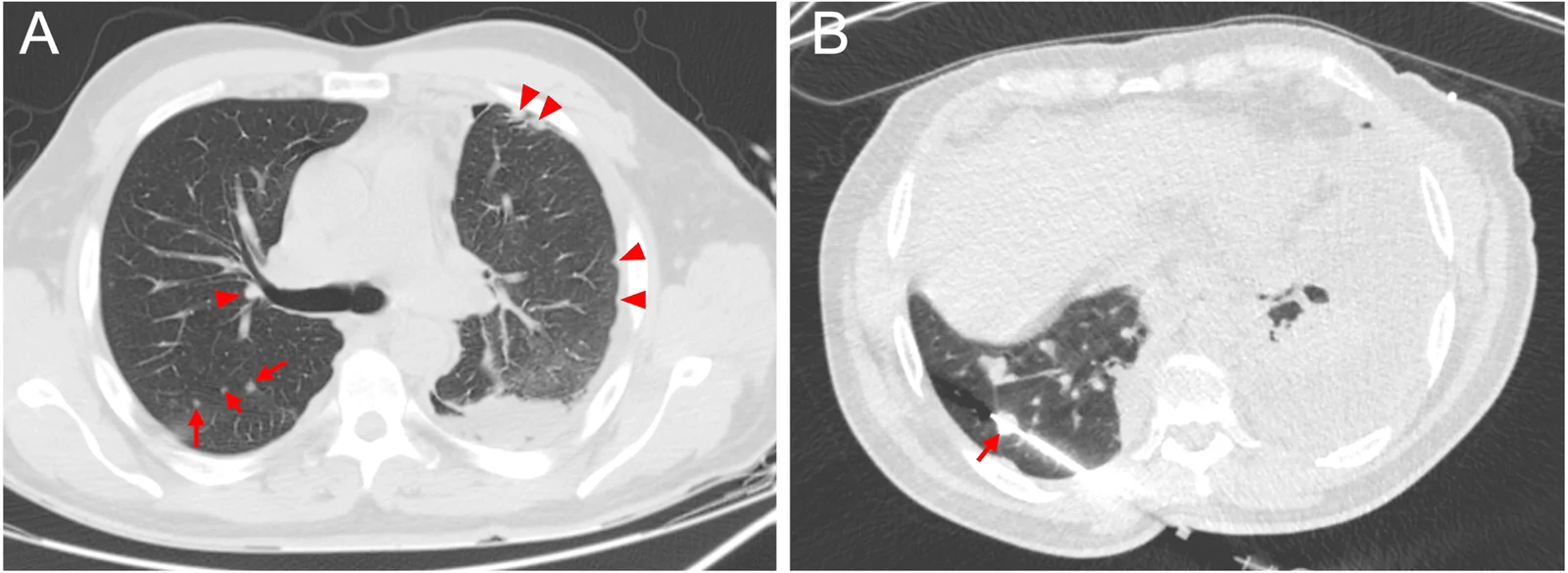
Radiologic findings of pulmonary metastatic disease. (A) Chest CT shows multiple intraparenchymal nodules (red arrows) and pleural-based soft-tissue nodules (red arrowheads), with associated pleural effusion, consistent with metastatic disease. (B) CT-guided core-needle biopsy demonstrates accurate needle placement within a pulmonary lesion (red arrow), enabling adequate sampling and subsequent histopathologic confirmation. CT: computed tomography.

Histologic examination demonstrated a high-grade carcinoma morphologically concordant with the previous sinonasal biopsy and resection specimen (Figure 7A). The metastatic tumor showed strong, diffuse expression of cytokeratins 7 and 8/18 and complete loss of INI1 nuclear expression, with retained staining in internal control elements (Figure 7B).

**Figure 7:**
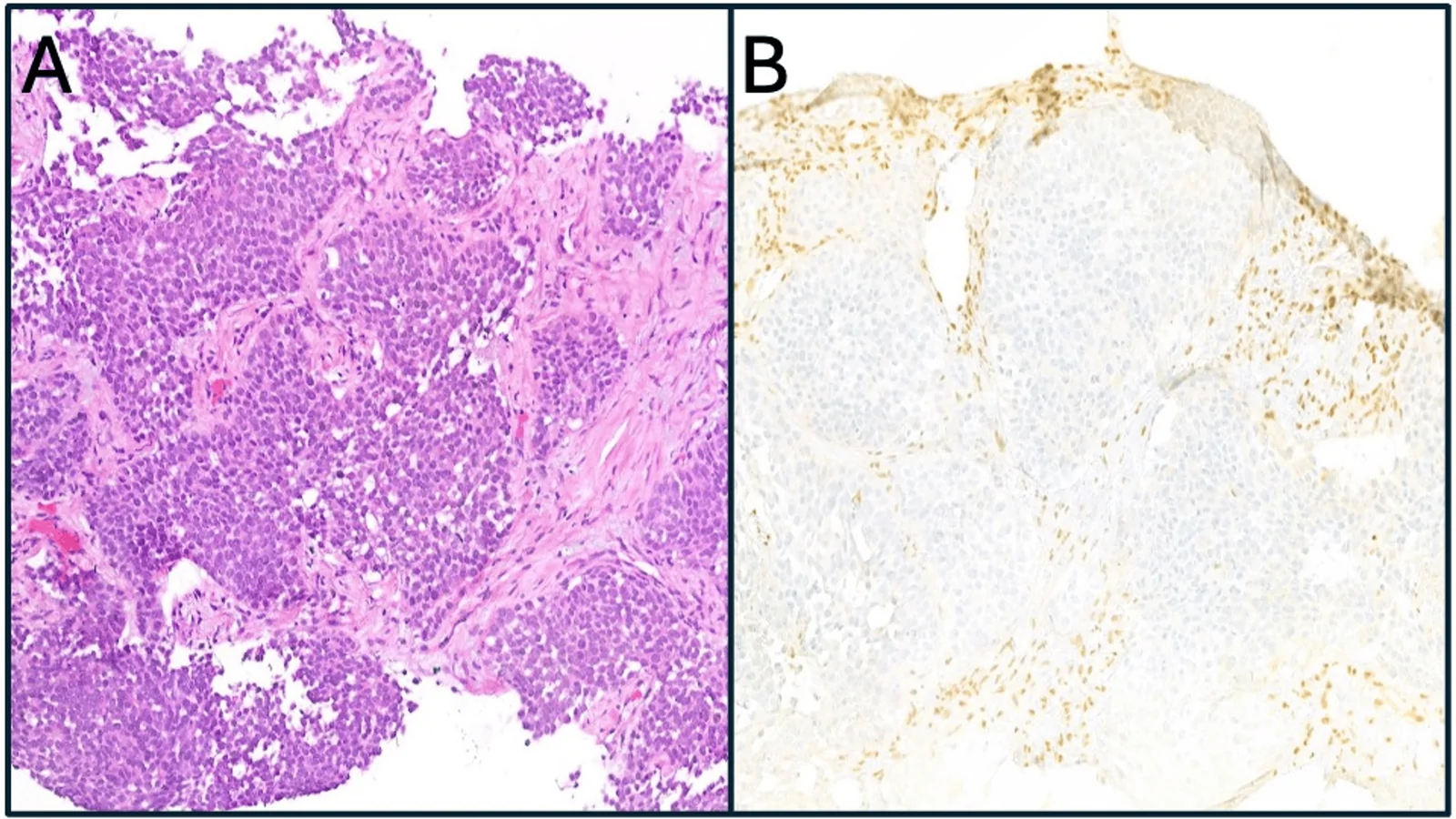
Histopathologic and immunohistochemical findings of the pulmonary metastasis. (A) Core biopsy of the pulmonary nodule demonstrates a poorly differentiated carcinoma morphologically concordant with the primary sinonasal tumor, showing solid nests and sheets of tumor cells separated by fibrotic stroma (hematoxylin and eosin, 20×). (B) INI1 immunohistochemistry shows complete loss of nuclear expression in the metastatic tumor cells, with retained nuclear staining in stromal and inflammatory cells serving as internal positive controls (20×). INI1: integrase interactor 1.

The tumor was negative for CD56, CD99, chromogranin, synaptophysin, desmin, and myogenin, while p53 demonstrated a wild-type staining pattern. These findings supported metastatic INI1-deficient carcinoma of sinonasal origin. The immunohistochemical findings from the primary biopsy and pulmonary metastasis are summarized in Table 1.

**Table 1:** Comparative immunohistochemical and in situ hybridization findings in the initial sinonasal biopsy and pulmonary metastasis. The primary sinonasal tumor and pulmonary metastasis demonstrated a concordant immunophenotype, including strong cytokeratin expression and complete loss of INI1 nuclear expression. Antibody clones and the EBER ISH probe are provided in parentheses. “Not performed” indicates that the corresponding study was not evaluated in that specimen. CK: cytokeratin; INI1: integrase interactor 1; NSE: neuron-specific enolase; CD: cluster of differentiation; EBER: Epstein-Barr virus-encoded RNA; ISH: in situ hybridization.

Marker (clone)	Initial sinonasal biopsy	Pulmonary metastasis
Pancytokeratin (AE1/AE3/PCK26)	Positive, strong and patchy	Not performed
CK7 (SP52)	Positive, patchy	Positive, strong and diffuse
CK8/18 (B22.1 & B23-1)	Positive, strong and diffuse	Positive, strong and diffuse
INI1 (MRQ-27)	Complete nuclear loss	Complete nuclear loss
p16 (E6H4)	Positive, strong and diffuse	Not performed
NSE (MRQ-55)	Positive, focal/patchy	Not performed
p53 (B653-11)	Not performed	Wild-type pattern
CD45 (RP2/18)	Negative	Not performed
CD56 (MRQ-42)	Not performed	Negative
CD99 (O13)	Negative	Negative
Chromogranin (LK2H10)	Negative	Negative
Synaptophysin (SP11)	Negative	Negative
S100 (rabbit polyclonal)	Negative	Not performed
CK5/6 (D5/16B4)	Negative	Not performed
p40 (ZR8)	Negative	Not performed
p63 (4A4)	Negative	Not performed
c-KIT (EP-10)	Negative	Not performed
EBER ISH (Probe 1)	Negative	Not performed
Desmin (DE-R-11)	Not performed	Negative
Myogenin (9FD5)	Not performed	Negative

Following confirmation of pulmonary metastatic disease, the patient received six cycles of pembrolizumab combined with cyclophosphamide, doxorubicin, and vincristine.

Comprehensive molecular profiling was later performed on archived tissue from the primary sinonasal resection by Caris Life Sciences (Irving, Texas, USA). Copy-number analysis identified a pathogenic SMARCB1 copy-number deletion, establishing the diagnosis of SMARCB1-deficient sinonasal carcinoma. The tumor was microsatellite stable and had a low tumor mutational burden of four mutations per megabase. Programmed death-ligand 1 (PD-L1) immunohistochemistry using the 22C3 clone was negative, with a combined positive score of 0. Human epidermal growth factor receptor 2 expression and Epstein-Barr virus-encoded RNA were also negative. No biomarker with sufficient clinical evidence for association with a specific approved therapy was identified.

These molecular results became available after systemic therapy had been initiated. Based on the integrated morphologic, immunohistochemical, and molecular findings, the original diagnosis was amended to SMARCB1-deficient sinonasal carcinoma, a molecularly defined subtype of SWI/SNF complex-deficient sinonasal carcinoma.

At the last documented follow-up, approximately one year after the initiation of systemic therapy and three years after the original diagnosis, follow-up fluorodeoxyglucose (FDG) positron emission tomography/computed tomography (PET/CT) demonstrated persistent bilateral FDG-avid pleural metastatic disease, including circumferential left pleural involvement encasing the lung, with a maximum standardized uptake value of 7.1 compared with 6.5 on the preceding study. A loculated left pleural effusion was also present. No focal residual FDG uptake was identified within the sinonasal surgical bed, and no new FDG-avid sites of disease were detected. These findings were interpreted as metabolically active but overall stable metastatic disease (Figure 8).

**Figure 8:**
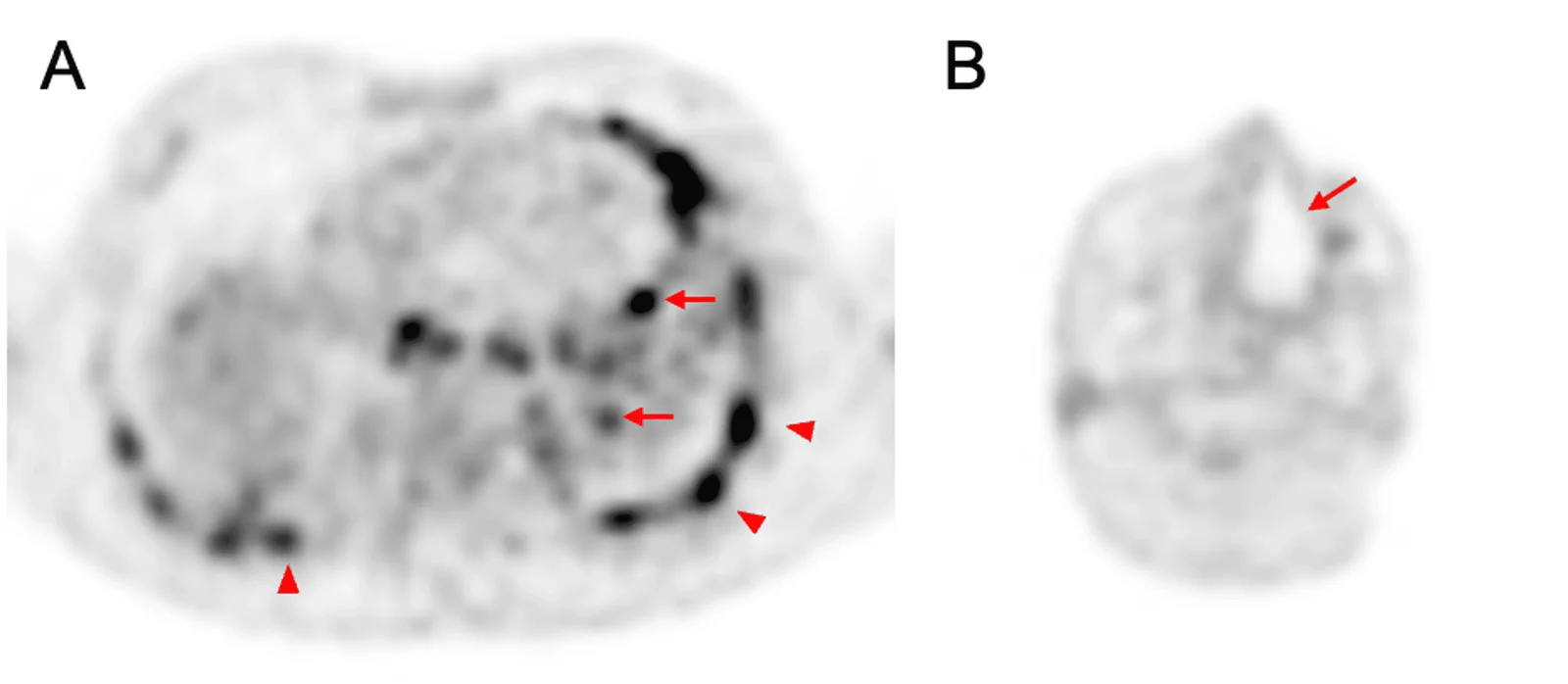
Follow-up fluorodeoxyglucose positron emission tomography/computed tomography findings. (A) Axial FDG PET image of the chest demonstrates FDG-avid intraparenchymal metastatic nodules (red arrows) and pleural-based metastatic deposits (red arrowheads), consistent with metabolically active thoracic disease. (B) Axial FDG PET image through the sinonasal region demonstrates the postoperative cavity in the left nasal cavity (red arrow), without focal residual FDG uptake in the surgical bed. FDG: fluorodeoxyglucose; PET/CT: positron emission tomography/computed tomography.

## Discussion

SMARCB1-deficient sinonasal carcinoma is a rare, highly aggressive, molecularly defined subtype of SWI/SNF complex-deficient sinonasal carcinoma that illustrates the increasing importance of molecular classification among poorly differentiated sinonasal tumors. Before recognition of SMARCB1-deficient carcinoma as a distinct entity, these neoplasms were frequently classified as SNUC, nonkeratinizing squamous cell carcinoma, neuroendocrine carcinoma, or other poorly differentiated malignancies [1-5]. In the present case, the tumor was initially classified as SNUC based on its high-grade morphology and epithelial immunophenotype; however, the concurrent complete loss of INI1 represented an important diagnostic clue to SWI/SNF complex deficiency. Subsequent identification of a pathogenic SMARCB1 copy-number deletion enabled definitive molecular classification as SMARCB1-deficient sinonasal carcinoma.

Histologically, SMARCB1-deficient sinonasal carcinomas commonly demonstrate basaloid or poorly differentiated morphology, with tumor cells arranged in solid nests, sheets, or trabeculae and variably exhibiting rhabdoid cytologic features [3-7]. Necrosis and brisk mitotic activity may be prominent. This morphologic spectrum creates substantial overlap with other small round blue cell and undifferentiated tumors of the sinonasal tract. The differential diagnosis includes conventional SNUC, neuroendocrine carcinoma, NUT carcinoma, SMARCA4-deficient sinonasal carcinoma, olfactory neuroblastoma, lymphoma, melanoma, and sarcomas. In this case, diffuse cytokeratin expression established epithelial differentiation, whereas negative neuroendocrine, hematolymphoid, and myogenic markers, together with the absence of S100 expression, argued against several major mimics. Strong, diffuse p16 expression should not independently be interpreted as evidence of human papillomavirus-associated disease, as p16 overexpression is not specific in this setting. Similarly, the negative Epstein-Barr virus-encoded RNA (EBER) result did not support an Epstein-Barr virus-associated carcinoma.

SMARCB1 encodes INI1, a core component of the SWI/SNF chromatin-remodeling complex. Loss of SMARCB1 function disrupts chromatin regulation and promotes oncogenesis through broad alterations in transcriptional control [3-5]. Genetic studies have demonstrated that homozygous SMARCB1 deletion is the most frequent mechanism underlying INI1 protein loss in these carcinomas, although hemizygous deletions and truncating alterations have also been identified [8]. Immunohistochemical loss of INI1, with retained staining in non-neoplastic internal controls, therefore provides a rapid and highly informative diagnostic finding. The complete absence of tumor-cell nuclear expression in both the sinonasal primary and pulmonary metastasis was particularly important in this case. Detection of a pathogenic SMARCB1 deletion in the archived primary tumor molecularly corroborated the immunophenotype and established a unifying diagnosis across specimens obtained approximately two years apart.

The clinical course also reflects the aggressive behavior reported for this entity. SMARCB1-deficient sinonasal carcinomas frequently present at an advanced local stage because of their anatomically confined site and nonspecific early symptoms [3-7,9,10]. The primary tumor in this patient measured at least 8 cm, involved the pterygoid muscle, and had positive medial and superior resection margins. Although neither lymphovascular nor perineural invasion was identified, the patient subsequently developed bilateral pulmonary nodules and a large pleural effusion. The initially sampled pleural fluid contained only rare, atypical epithelial cells and was insufficient for a definitive malignant diagnosis. In contrast, adequate core sampling of a right pulmonary nodule demonstrated a carcinoma morphologically and immunophenotypically concordant with the sinonasal primary. This distinction is clinically important because cytologically paucicellular effusions may be nondiagnostic, whereas tissue sampling can permit comparison of morphology and lineage-defining biomarkers.

Because of its rarity, no universally accepted treatment algorithm has been established for SMARCB1-deficient sinonasal carcinoma. Management is generally multimodal and may include surgical resection followed by radiation, with concurrent or sequential platinum-based chemotherapy depending on disease extent and resectability [5,9,10]. The patient underwent an extensive surgical resection followed by 7,200 cGy of radiotherapy and nine cycles of platinum-based chemotherapy. Retrospective series suggest that aggressive multimodal therapy may provide locoregional control, but recurrence and distant metastasis remain common, and conclusions are limited by small sample sizes, treatment heterogeneity, and selection bias [9,10].

Evidence supporting systemic therapy for recurrent or metastatic disease remains particularly limited. Published reports have described immune checkpoint inhibitors in selected patients with SMARCB1-deficient sinonasal carcinoma, but the small number of cases precludes reliable estimates of response or identification of predictive biomarkers [11]. In the present case, the patient began pembrolizumab in combination with cyclophosphamide, doxorubicin, and vincristine before the molecular profiling results became available. Subsequent testing demonstrated that the tumor was microsatellite stable, had a low tumor mutational burden, and showed a PD-L1 combined positive score of 0. Follow-up FDG PET/CT demonstrated persistent metabolically active pleural metastatic disease without interval progression or new sites of disease. Because pembrolizumab was administered as part of a multidrug regimen, the contribution of any individual agent cannot be determined. Moreover, disease stability in a single patient should not be interpreted as evidence that conventional immune-response biomarkers are irrelevant in this tumor type.

SMARCB1 loss also provides a potential therapeutic rationale for targeting enhancer of zeste homolog 2 (EZH2), a catalytic component of the polycomb repressive complex 2 that functionally opposes SWI/SNF activity. Tazemetostat, an EZH2 inhibitor, has demonstrated clinical activity in advanced epithelioid sarcoma with INI1 loss [12]. However, those data cannot be directly extrapolated to SMARCB1-deficient sinonasal carcinoma, and the role of EZH2 inhibition in this disease remains investigational. Collaborative molecularly informed trials will be necessary to evaluate such targeted approaches in this exceptionally uncommon malignancy. Pulmonary metastasis of SMARCB1-deficient sinonasal carcinoma has previously been described as a diagnostic pitfall, emphasizing the importance of clinical history, morphologic comparison with the primary tumor, and INI1 immunohistochemistry when evaluating a poorly differentiated carcinoma in the lung [13].

This case highlights three practical lessons. First, complete INI1 loss should prompt consideration of SMARCB1-deficient carcinoma whenever a poorly differentiated sinonasal malignancy is encountered. Second, re-evaluation of an earlier nonspecific diagnosis may be warranted when new immunohistochemical or molecular information becomes available. Finally, comparison of the primary and metastatic tumors, including morphology, epithelial markers, and INI1 expression, can establish the origin of distant disease and directly affect classification, prognostication, and treatment planning.

## Conclusions

SMARCB1-deficient sinonasal carcinoma should be considered in the differential diagnosis of any poorly differentiated sinonasal malignancy, particularly when the initial findings suggest SNUC. Complete loss of INI1 expression, supported when available by molecular confirmation of SMARCB1 alteration, enables accurate classification and distinction from morphologically overlapping entities. In this patient, concordant morphology and INI1 loss in the primary tumor and pulmonary metastasis established the origin of the distant disease approximately two years after the original diagnosis. This case underscores the aggressive behavior of this rare carcinoma, the value of adequate tissue sampling at recurrence, and the importance of reassessing earlier diagnoses as molecular classifications evolve. Although the patient had persistent metabolically active metastatic disease without interval progression at the last documented follow-up following combination systemic therapy, the optimal management of metastatic SMARCB1-deficient sinonasal carcinoma remains undefined and requires further collaborative investigation.
